# Cyanobacteria and Cyanotoxins Occurrence and Removal from Five High-Risk Conventional Treatment Drinking Water Plants

**DOI:** 10.3390/toxins7062198

**Published:** 2015-06-12

**Authors:** David C. Szlag, James L. Sinclair, Benjamin Southwell, Judy A. Westrick

**Affiliations:** 1 Chemistry Department, Oakland University, Rochester, MI 48309, USA; E-Mail: szlag@oakland.edu; 2 Office of Groundwater and Drinking Water, Technical Support Center, USEPA, Cincinnati, OH 45268, USA; E-Mail: sinclair.james@epa.gov; 3 Environmental Analysis Laboratory, Lake Superior State University, Sault Ste. Marie, MI 49783, USA; E-Mail: bsouthwell@lssu.edu; 4 Lumigen Instrument Center, Department of Chemistry, Wayne State University, Detroit, MI 48202, USA; E-Mail: westrick@chem.wayne.edu

**Keywords:** cyanobacteria, microcystin, anatoxin-a, cylindrospermopsin, conventional drinking water treatment

## Abstract

An environmental protection agency EPA expert workshop prioritized three cyanotoxins, microcystins, anatoxin-a, and cylindrospermopsin (MAC), as being important in freshwaters of the United States. This study evaluated the prevalence of potentially toxin producing cyanobacteria cell numbers relative to the presence and quantity of the MAC toxins in the context of this framework. Total and potential toxin producing cyanobacteria cell counts were conducted on weekly raw and finished water samples from utilities located in five US states. An Enzyme-Linked Immunosorbant Assay (ELISA) was used to screen the raw and finished water samples for microcystins. High-pressure liquid chromatography with a photodiode array detector (HPLC/PDA) verified microcystin concentrations and quantified anatoxin-a and cylindrospermopsin concentrations. Four of the five utilities experienced cyanobacterial blooms in their raw water. Raw water samples from three utilities showed detectable levels of microcystins and a fourth utility had detectable levels of both microcystin and cylindrospermopsin. No utilities had detectable concentrations of anatoxin-a. These conventional plants effectively removed the cyanobacterial cells and all finished water samples showed MAC levels below the detection limit by ELISA and HPLC/PDA.

## 1. Introduction

Cyanobacteria, also known as blue-green algae, are photosynthetic bacteria that can live in many types of water. Rapid, excessive cyanobacteria growth, often referred to as a “bloom”, is linked to eutrophication and high water temperatures. Many genera of cyanobacteria are known to produce toxins. These toxins (cyanotoxins) make up a large group of chemical compounds that differ in their molecular structure and toxicological properties. They are generally grouped into major classes according to their toxicological targets: cell, liver, nervous system, skin, and tumor promotion. Microcystins are hepatotoxins commonly produced by the cyanobacteria genera *Anabaena*, *Microcystis*, *Oscillatoria*, *Planktothrix*, *Nostoc*, and *Hapalosiphon*. Cylindrospermopsin is a hepatotoxin and cytotoxin produced by the filamentous cyanobacteria *Aphanizomenon* and *Cylindrospermopsis*. Both microcystin-LR [1] and cylindrospermopsin [2] are suspected tumor promotors. Anatoxin-a is a neurotoxin produced by the cyanobacteria *Aphanizomenon*, *Anabaena*, and *Oscillatoria*. Common freshwater cyanobacteria genera like *Microcystis*, *Planktothrix*, *Aphanizomenon* and *Anabaena* contain many species and genotypes that may be both toxic and capable of forming blooms and also may cause problems not related to toxicity in water bodies used as drinking water sources [3]. A significant feature of these blooms is that their cyanotoxin production is highly variable. A single bloom may contain multiple types of cyanotoxins because a bloom may have more than one toxin-producing genus [4] and/or potentially one genus may produce more than one toxin [5]. However, occurrence of a cyanobacteria bloom does not necessarily mean there is a cyanotoxin problem. Multiple genotypes of cyanobacteria can exist in a single bloom, and some produce toxins while others do not. Even genotypes or species that can produce toxins do not always produce the toxins. Under some conditions toxic genotypes will not produce toxins at all. The environmental conditions that trigger or inhibit production of cyanotoxins remain poorly understood and remain an active area of research. Another feature common to cyanobacterial blooms is the formation of surface scums or bands of high cell concentration in the water column. Surface scums are often blown by the wind into bays and areas with poor water circulation allowing cyanobacteria and cyanotoxins to build up to very high concentrations.

There are numerous studies that have surveyed virtually all regions of the planet for the occurrence of cyanobacteria and cyanotoxins. WHO (1999) [5], and Fristachi *et al*. [6] provide an overview of the worldwide occurrence studies. Within the U.S. and Canada there are numerous reports from state and local health agencies that have not been published in the peer-reviewed literature but are available through websites and bulletins. The occurrence of microcystin producers and microcystins dominate these reports. Very few studies have investigated cylindrospermopsin or anatoxin-a in North America. Graham *et al*. [7] provides a detailed survey of cyanobacteria and cyanotoxins, including seven microcystin congeners, anatoxin-a, cylindrospermopsin, lyngbya toxin-a, and nodularin in reservoirs and lakes in the Midwestern U.S. Microcystin is the most frequently observed toxin in this study.

As of 2015, insufficient epidemiological data are available to develop a guideline value or standard for lifetime exposure to any of the cyanotoxins. High acute exposures to microcystins can cause gastroenteritis and liver damage [5]. Data and studies on chronic human exposure to microcystins are sparse. Studies that have come out of China supporting a link between elevated cancer incidence and exposure to microcystins include those by Zhou *et al*. (2000) [8] and works by Yu (2001) [9]. No information is available on the carcinogenicity of cylindrospermopsin in humans, and no definitive cancer studies of purified cylindrospermopsin have been conducted in animals. Falconer and Humpage (2006) observed a tenuous link between cylindrospermopsin exposure and tumor growth in a mouse study but the study lacked statistical power [10]. No studies link anatoxin-a to chronic health effects. The provisional WHO guideline value for microcystin LR is based on doses from short-term mouse studies using the no adverse effect level (NOAEL) methodology [11]. A tolerable daily intake (TDI) of 0.04 μg/kg body weight per day was derived from the 40 μg/kg NOAEL body using an uncertainty factor of 1000 (10 for intraspecies variation ×10 for interspecies variation and ×10 for less than-lifetime study). For drinking water, the WHO provisional guidance value (PGV) defines concentrations considered safe for lifetime consumption of 2 L of drinking-water per (60 kg person × day). Based on the described approach, the World Health Organization (WHO) through its updated 2011 Guidelines for Drinking-Water Quality [12] has recommended a provisional guidance value of 1 ug/L (1 ppb) total microcystin-LR in drinking water. It should be noted that this value includes free and cell-bound toxin and is for chronic exposure. Microcystins are the most widely researched group of toxins with microcystin-LR being the most frequently encountered as well as being one of the more toxic congeners. Consequently values developed for microcystin LR are generally considered to be conservative with respect to protecting public health. Some countries have used slightly different factors or included all microcystin congeners, but most worldwide guidelines range between 1.0 and 1.5 ug/L microcystin LR or LR equivalents. Some local health jurisdictions, areas of Scotland, as an example, allow short-term exposures to microcystins to exceed the WHO provisional guideline (Suggested No Adverse Effects: 24-h 12.0 ug/L and 7-day 6.0 ug/L microcystin-LR) [13]. Chorus [14] has compiled an unofficial partial list of provisional guidance values (PGVs), health alert levels (HALs) and standards from across the world. This compilation also includes values from a few countries that have set PGVs for cylindrospermopsin, anatoxin-a, and saxitoxins. PGVs for cylindrospermopsin range from 1–15 ug/ L and PGVs for anatoxin-a range from 1–6 ug/L.

Currently, there are no U.S. federal regulatory guidelines or standards for cyanobacteria or their toxins in drinking water. Many states and local health authorities rely on guidelines published by the WHO or have derived their own guidelines to support public health decision-making. The Safe Drinking Water Act (SDWA) requires the U.S. Environmental Protection Agency (USEPA) to publish a list of unregulated contaminants that are present or expected to be detected in public water systems. These chemicals are on the drinking water Contaminant Candidate List (CCL). The CCL itself does not impose any requirements on public water systems. Instead, USEPA uses it to prioritize research efforts to help determine whether a contaminant has sufficient data to meet regulatory determination criteria specified in the SDWA. Freshwater cyanobacterial toxins were initially named to the drinking water Contaminant Candidate List (CCL) in 1998 by the Environmental Protection Agency (USEPA) based on insufficient data concerning toxicity, occurrence, and susceptibility to treatment (Table 2 of 63 FR 10273 [15]). In 2001 the US priority list of freshwater algal toxins included four of the more than eighty variants of microcystin (RR, LR, YR, and LA), cylindrospermopsin, and anatoxin-a. In 2012, three cyanotoxins remain listed on the CCL 3: anatoxin-a, microcystin-LR, and cylindrospermopsin. The USEPA did not implement the Unregulated Contaminant Monitoring Rule (UCMR) to more thoroughly assess the occurrence of cyanotoxins through the UCMR 3 initiated in 2012 (EPA, UCMR 3).

For the drinking water industry, the casual chain follows that when toxin producing genera are present in the source there is a risk that toxins will be present in the raw water; when toxins are present in the raw waters, there is a risk for toxins to also be present in finished drinking water. Drinking water utilities must manage this risk with appropriate responses that balance consumer safety, staff resources, economics, and the inherent variability of cyanobacteria blooms. Over the past two decades, several Risk Management Frameworks (RMFs) have been proposed by Burch [16,17], the WHO [5] and van Baalen and Du Preez [18]. All of these frameworks share a similarity in that progressive responses are based on parameters directly linked to toxic cyanobacteria such as cell numbers, chlorophyll-a, biovolume, biomass, and /or direct measurement of the cyanotoxin. In implementing any risk management plan a utility would assess its resources, treatment processes, source water(s) and geography, modify the plan for local conditions, and then implement it. In 1993 and again in 1999, the World Health Organization (WHO) presented a framework for cyanobacteria and toxin monitoring that have become the template for system specific risk management plans known as water safety plans by the WHO [5]. The original WHO risk management framework included three levels: a Vigilance Level, an Alert Level 1 and an Alert Level 2, with corresponding responses. The Vigilance Level would be achieved when cyanobacteria were detected at low concentrations. The main responses would be an increase in monitoring of the source water and monitoring of the raw water at the intakes by microscopy. Alert Level 1 would be achieved when the cyanobacterial cell concentration exceeded 2000 cyanobacteria cells/mL, or the chlorophyll-a concentration of the raw water exceeded 10 µg/L. Calculations showed that at these concentrations it was possible, but not necessarily likely, that the WHO provisional guideline, 1 ug/L, for microcystin-LR would be exceeded in the raw water. At this alert level the main responses could include increased monitoring frequency, cyanotoxin analysis, altering intake depths or locations, an assessment of the drinking water treatment barriers for cyanobacteria and cyanotoxin removal and communication with health officials and the public. Alert Level 2 would be reached when the cyanobacterial cell concentration exceeded 100,000 cells/mL, or the chlorophyll-a concentration of the raw water exceeded 50 µg/L and the cyanobacteria are shown to be toxic. The main actions during this alert level would include continued monitoring, treatment optimizations (often powdered activated carbon: PAC), consideration of alternative water supplies, and increased communication with health officials and the media. The WHO Alert Level Framework was useful, but recently the WHO has recognized limitations in a prescriptive AL risk management approach and has promoted an adaptive and holistic approach that is based on the Hazard Analysis and Critical Control Point (HACCP) approach used in the food industry. This approach recognizes that each utility is unique and that the levels and responses should be adjusted to each DWTP. Chorus [14] provides an overview of this approach and web based step by step guidance on water safety plans (WSPs).

Key information for implementation and discussion of the HAACP and water safety plan approach are the levels of cyanobacteria and cyanobacterial toxins in the raw water and finished water at each DWTP. The number of DWTP studies which measured cyanobacterial and toxins in raw and finished waters is limited. Hoeger *et al*. [19] provide a partial review and summary of world-wide drinking water treatment plant (DWTP) performance. Karner *et al*. [20] surveyed microcystin occurrence in raw and finished water from five utilities which used source water from two lakes. Bloom levels were visually noted. Lahti *et al*. [21] analyzed raw and finished water samples for microcystin and also determined the composition of cyanobacterial biomass by microscopy. Of particular relevance to this study are the major evaluations of North American DWTPs by Carmichael [22] and Robert *et al*. [23]. Carmichael [22] conducted a survey of 45 utilities across the U.S. and Canada for two years during bloom conditions when cyanobacteria reached or exceeded 2000 cells/mL. Microcystin concentrations were measured in raw and finished waters. All of these studies only investigated the occurrence of microcystin. Anatoxin-a has been rarely detected in North American drinking water sources and in general at low concentrations according to Roberts *et al*. [23] and Boyer *et al*. [24]. More recently Graham *et al*. [7] found that 30% of the lakes sampled in their Midwest U.S. survey contained anatoxin-a with concentrations ranging from 0.05–9.5 ug/L. The main concern with cylindrospermopsin-producing blooms, in contrast with microcystin-producing blooms, is that at different stages of a *Cylindrospermopsis* bloom, extracellular cylindrospermopsin concentrations can be substantial and range from 19 to 98% of the total amount in water [25,26]. Given the increasing presence and abundance of cylindrospermopsin and anatoxin-a-producing genera along with widespread occurrence of microcystin producers, the USEPA sought updated and expanded data for all priority toxins including the Microcystins, Anatoxin-a, and Cylindrospermopsin (MAC) [27]. Recently Zamyadi *et al*. evaluated all processes in a conventional DWTP for the removal of cyanobacteria and cyanotoxins [28]. This study intensively monitored the DWTP processes three times over 1–3 day intervals in 2008, 2009, and 2010. In 2008 and 2009 only microcystin-LR-eq were monitored. In 2010 multi-toxin LC/MS/MS method was used to identify priority microcystin congeners and cylindrospermopsin. On one occasion, traces of cylindrospermopsin were detected.

The aim of the present study was to collect concentration data regarding the MAC toxins in raw water and finished drinking water (clear well effluent) and the abundance of potential toxin producers during non-bloom and bloom conditions. Among the questions we hoped to answer from this study were:

- What is the likelihood of encountering detectable MAC toxins in different geographic areas of the U.S.?
- How do MAC concentrations from this study compare to levels found elsewhere?
- How effective are conventional DWTPs at removing MAC toxins and cyanobacterial cells?
- How do microcystin concentrations compare to the WHO provisional guideline level and to other proposed guidelines for cylindrospermopsin and anatoxin-a?
- How do measured MAC concentrations correlate with the cyanobacterial cell density alert level framework as described in Chorus and Bartram [5]?

## 2. Results and Discussion

A key element of a management program or water safety plan based on the WHO templates is the microscopic identification and enumeration of the cyanobacteria present in the raw water. In this study, simplified, rapid microscopic methods were used to estimate the cyanobacterial cell numbers and genus composition, at the genus level, in the raw and finished drinking water at five “high risk” DWTPs located in five states distributed across the U.S. Two graphs are presented for each DWTP. The first graph contains total algae and cyanobacteria, total cyanobacteria, and microcystin plotted on the secondary *y*-axis. Cylindrospermopsin was not plotted because it was only detected in one raw water sample. Anatoxin-a was not plotted because it was never detected. On the figures, microcystin levels below the 0.05 ug/L detection of the ELISA kit were plotted as zeros. Total algae were counted as naturally occurring colonies or cell aggregations which were referred to as units, whereas cyanobacteria were counted as individual cells or converted to individual cells. The second graph for each DWTP presents the cell concentration of the dominant potential toxin-producing cyanobacteria genera.

The goal of this study was to present a snapshot of the range of occurrence and concentration of MAC toxins and both total and potential toxin-producing cyanobacteria and to understand how conventional DWTPs performed over a 12–16 week spring-summer observation period. These five DWTPs, located in California, Texas, Oklahoma, Florida, and Vermont, were known to have potential toxin-producing cyanobacteria in their source waters and to experience a high frequency of blooms based on the author’s observations, reports in the literature, or media reports. During the 12–16 week observation period, four of the five DWTPs in California, Texas, Florida (River source), and Oklahoma, total cyanobacteria cell numbers often exceeded the WHO AL 1 for cyanobacterial cells. The California DWTP raw water exceeded the WHO AL 2 on two occasions and the Texas DWTP exceeded the WHO AL 2 on one occasion. The concentrations of MAC toxins, however, were extremely low or below detection with only 1 out of the 71 (~1%) raw water samples exceeding the WHO PGV of 1 ug/L microcystin-LR.

General observations for cyanotoxins included:

- Microcystins were observed frequently in the raw water at low concentrations between 0.05 and 0.25 ug/L.
- Cylindrospermopsin was only detected in one raw water sample (Oklahoma at 0.41 ug/L in the May 2 sample).
- Anatoxin-a was not detected in any raw water sample.
- No MAC cyanotoxins were detected in any finished drinking water.
- There was no correlation between numbers of toxin-producer cyanobacteria and levels of toxins found.

The single detection of cylindrospermopsin was unexpected given the low numbers of potential cylindrospermopsin-producers present in that sample, and there were no detections of anatoxin-a in the raw water samples. It is possible that the availability and the increased sensitivity of the ELISA for microcystin compared to using only a less sensitive HPLC/PDA method for cylindrospermopsin and anatoxin-a may have skewed the frequency of detection results. Of the 43 total detections of microcystin by both ELISA and HPLC, 88% were below the detection limit for HPLC/PDA of 0.25 ug/L.

### 2.1. Individual Sites

#### 2.1.1. California Plant

**Figure 1:**
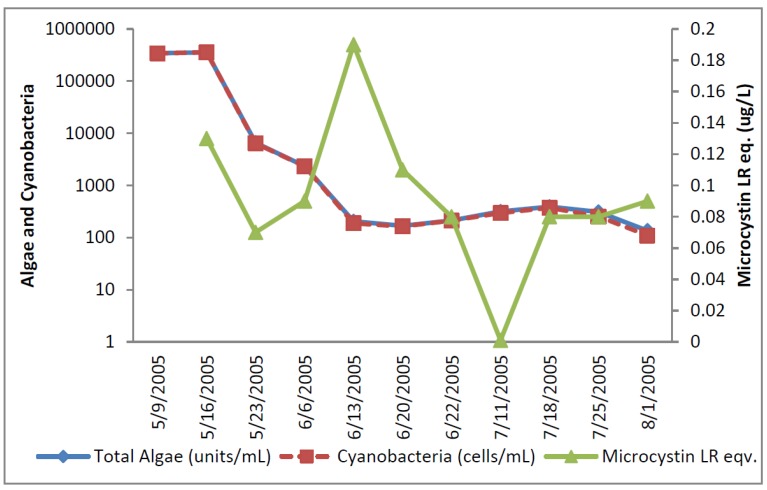
California algal density and ELISA microcystin concentration in raw water.

**Figure 2:**
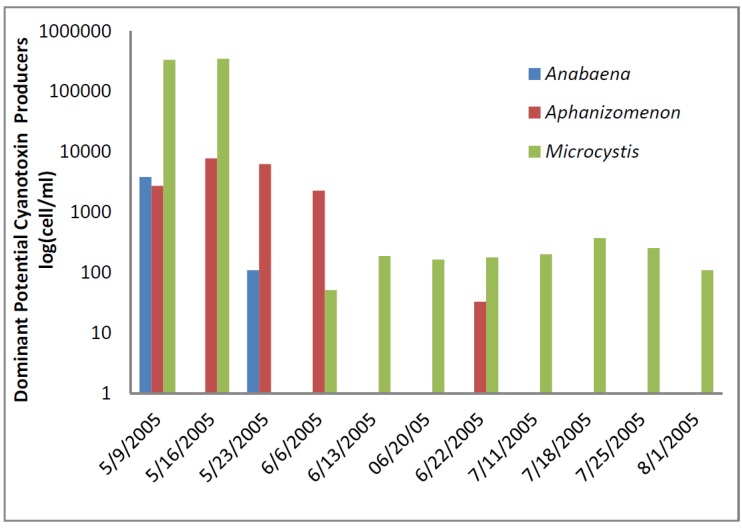
California site cyanobacteria potential producers of individual toxins.

The greatest number of cyanobacteria found in any raw water occurred at the California DWTP (Figure 1). More than 300,000 *Microcystis* cells/mL were found on 9 May 2005 and 16 May 2005, which greatly exceeded the WHO AL 1 and WHO AL 2 monitoring framework thresholds of 2000 cells/mL and 100,000 cells/mL respectively. On two other occasions AL 1 was exceeded. On these dates, small colonies of *Microcystis* (Figure 2) accounted for almost all of the total cyanobacteria in these samples. Mid-summer samples from the California site showed that both total cyanobacteria and toxin-producing cyanobacteria declined to less than 1000 cells/mL. Figure 2 shows that through most of the sample period potential microcystin-producers outnumbered potential producers of cylindrospermopsin or anatoxin-a at the California site. No algal/cyanobacteria cells were found in any finished drinking water sample except for 1 August 2005 when 80 *Oscillatoria* cells/mL were found. The filaments that broke through the filter consisted of approximately 30 cells/unit. These results show that there was as much as 5.5 log removal of total cyanobacteria and potential toxin producers by water treatment (Table 1).

Figure 1 shows that low levels of microcystin, determined by ELISA, were detected in all but the 11 July 2005 sample. The highest amount of microcystin detected by ELISA was 0.19 ug/L in the 6 June 2005 sample, which did not coincide and lagged the highest densities of *Microcystis* cells (Figure 1). This same sample, from 6 June 2005, was found to contain microcystin-LR at 0.79 ug/L when determined by HPLC/PDA. This was the only discrepancy between the ELISA (Envirologix Inc., Portland, OR, USA) and HPLC/PDA (Waters Corporation, Milford, CT, USA) analyses at the California site. HPLC/PDA analysis did not detect microcystin in any other sample, or cylindrospermopsin or anatoxin-a in any sample of raw water. No toxins were detected by ELISA or HPLC in any finished drinking water sample.

#### 2.1.2. Texas Plant

**Figure 3:**
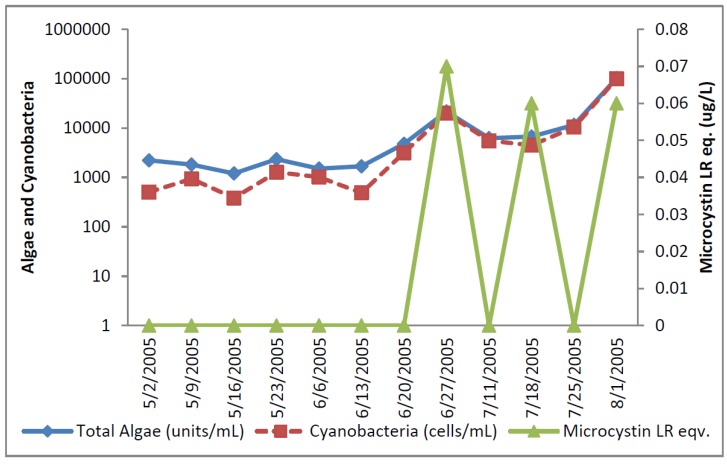
Texas algal density and ELISA microcystin concentration in raw water.

**Figure 4:**
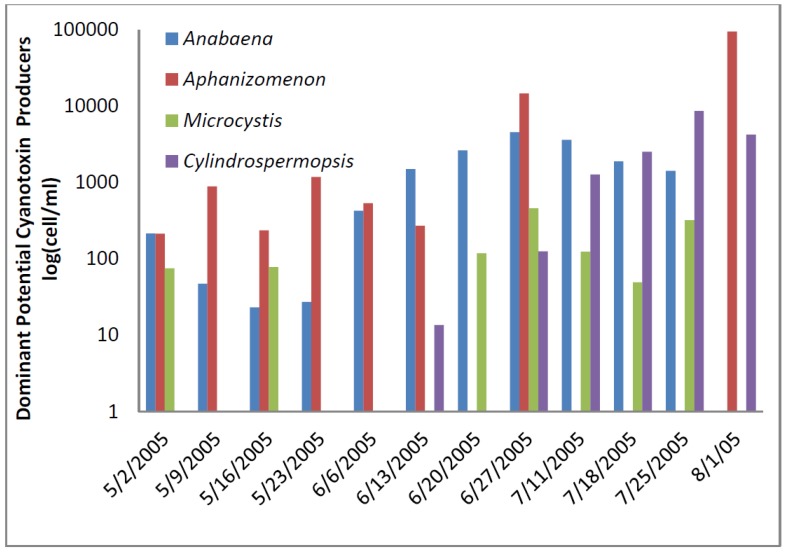
Texas toxin-producing cyanobacteria.

At the Texas site, potential toxin-producing cyanobacteria exceeded the AL 1 of 2000 cells/mL toward the end of sampling period five times (Figure 3) and AL 2 once. Total potential toxin-producers were well below 2000 cells/mL at the beginning of sampling period and generally represented less than half of the total cyanobacteria at that time. Both total cyanobacteria and total potential toxin-producing cyanobacteria increased over time so that toward the end of sampling potential toxin-producers accounted for nearly all of the cyanobacteria present. *Cylindrospermopsis* exceeded 2000 cells/mL on 18 July and 25 July and accounted for more than half of the total cyanobacteria on those dates. By 1 August 2005 the bloom became dominated by *Aphanizomenon*, which may produce cylindrospermopsin and anatoxin-a. It was present at 4700 cells/mL. The abundance of *Cylindrospermopsis* and *Aphanizomenon* near the end of sampling was responsible for the large numbers of potential cylindrospermopsin and anatoxin-a-producers at these times (Figure 4). *Anabaena* increased during the initial sampling period and exceeded 2000 cells three times, and declined after 18 July 2005 (Figure 4). It is a potential microcystin and anatoxin-a-producer.

Microcystin detected by ELISA was observed in three samples at levels slightly above the detection limit during the latter part of the sampling period (Figure 4). HPLC/PDA did not detect microcystin, cylindrospermopsin, or anatoxin-a in any sample despite high densities of potential cylindrospermopsin and anatoxin-a-producers.

In the Texas DWTP finished water, 1 total algal unit/mL was detected in the 2 May, 9 May, 23 May, and 18 July 2005 samples. Additionally, less than 80 cells /mL of *Cylindrospermopsis*, *Aphanizomenon* and *Anabaena* were observed in the 11 July 2005 sample. No microcystin, cylindrospermopsin or anatoxin-a were observed in any distribution system sample. The log removal of total cyanobacteria by treatment was up to 4.0 log and about the same for the toxin-producers (Table 1).

**Table 1:** Range of cell removal by water treatment for total cyanobacteria and toxin-producers.

Location	Total Cyanobacteria (Range of cell removal (log_10_))	Toxin Producers (Range of cell removal (log_10_))
California	1.5 to >5.5	1.5 to >5.5
Oklahoma	1.6 to >3.4	0.2 to >3.2
Vermont	>2.5 to 3.1	* to >2.2
Texas	>2.8 to >4.0	>1.6 to >4.0
Florida (both sources)	1.6 to 3.8	1.6 to 3.3

* log removal cannot be determined. Toxin producer numbers were very low in the raw water, and not detected in the finished water.

#### 2.1.3. Florida DWTP

**Figure 5:**
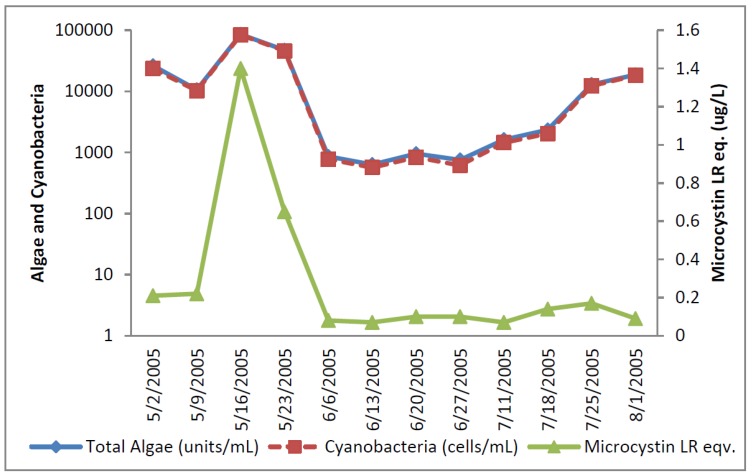
Florida River algal density and ELISA microcystin concentration in raw water.

**Figure 6:**
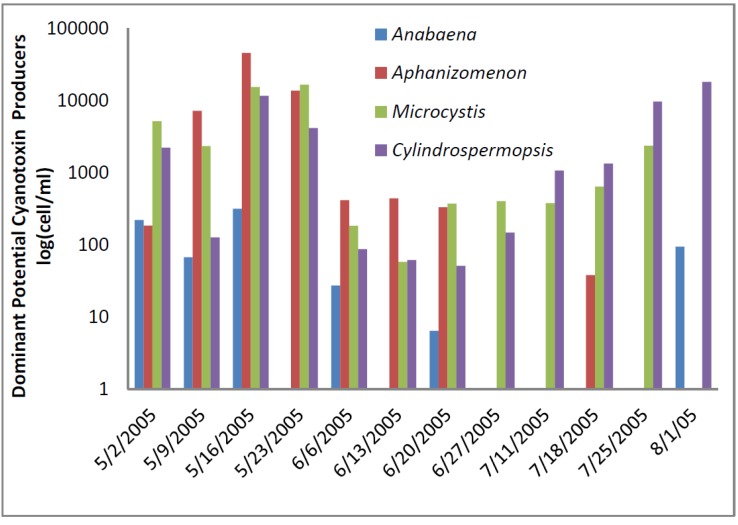
Florida River toxin-producers.

The Florida Plant removed water from the river and pumped into a reservoir. Since the reservoir had longer than one day retention time, the reservoir was included as a second source to the utility. The Florida River exceeded the WHO Alert Level 1 monitoring level for eight of the raw water samples analyzed for cyanobacterial cells (Figure 5). Total potential toxin-producing cyanobacteria exceeded WHO Alert Level 1 for cell densities on 16 May, 23 May, and 23 July 2005, with the greatest number of 43,000/mL occurring on 16 May 2005 (Figure 6). *Aphanizomenon* was dominant. Potential toxin-producers were somewhat lower than total algal numbers during sampling but followed the same density trends in most samples. Potential cylindrospermopsin and anatoxin-a-producers increased to their highest numbers on 16 May 2005, declined through 27 June 2005 and increased thereafter. Potential microcystin-producers varied between 15,000 and 16,000 cells/mL. The potential microcystin-producers on this date consisted of *Microcystis* and *Oscillatoria*.

The ELISA microcystin-LR equivalent analyses increased up to 1.41 ug/L microcystin-LR equivalents on 16 May 2005 before declining in later samples to levels below 0.2 ug/L. The 16 May 2005 raw water sample was the only sample in the entire study that exceeded the WHO guideline level of 1 ug/L for microcystin-LR in drinking water. Microcystin, cylindrospermopsin, and anatoxin-a were not detected by HPLC/PDA in any raw water sample. Microcystins, cylindrospermopsin, and anatoxin-a were not detected by HPLC/PDA or ELISA in any finished water sample.

The first eight finished water samples contained between 2 and 11 total and potential toxin-producer cyanobacteria per mL in the finished water, except for 23 May 2005 when 340 cells/mL of *Anaebena* and 1260 cells/mL of *Aphanizomenon* were present. After the eighth sample, finished water samples contained 0 to 20 cells of total cyanobacteria/mL. Water treatment reduced total cyanobacteria by as much as log 3.7 and toxin-producers by as much as log 3.3 (Table 1).

**Figure 7:**
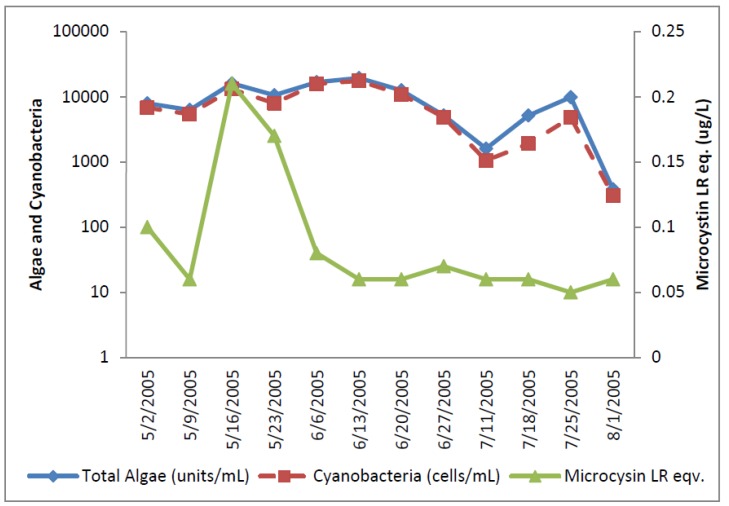
Florida Reservoir Cyanobacteria and Microcystin.

**Figure 8:**
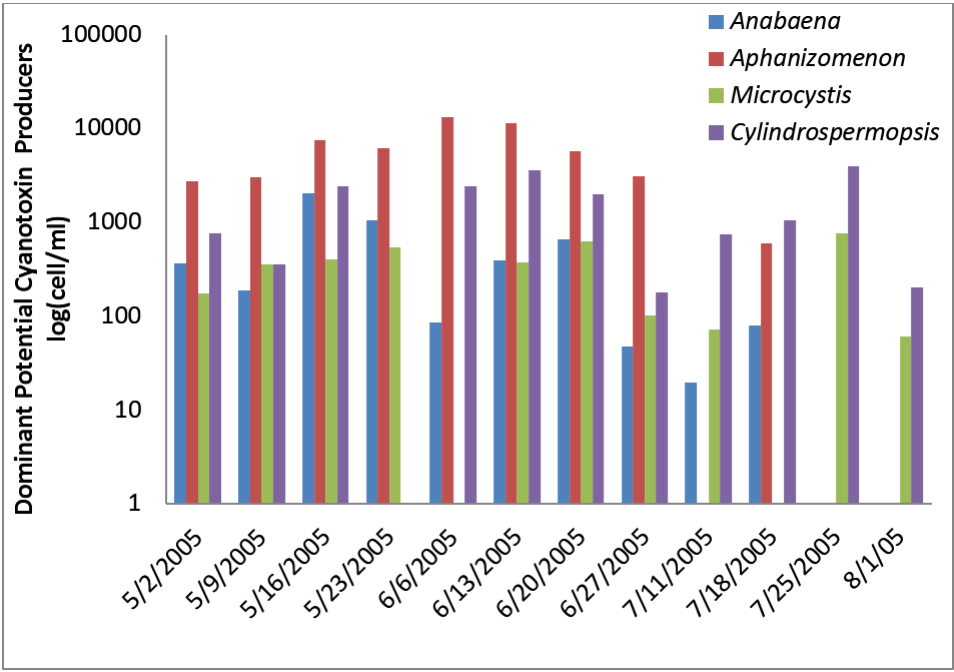
Florida Reservoir individual toxin producers.

The total cyanobacteria exceeded AL 1 11 times in the reservoir. The potential toxin-producer cyanobacterial units were always lower than the total cyanobacteria in the Florida reservoir samples (Figure 7 and Figure 8). The dominant genus was *Aphanizomenon*. *Microcystis* genera were uncommon and almost disappeared late in the sample period.

Microcystin as determined by ELISA was found in low concentrations in all samples except one. Microcystin, cylindrospermopsin, and anatoxin-a were not detected in the Florida reservoir samples by HPLC.

#### 2.1.4. Oklahoma

**Figure 9:**
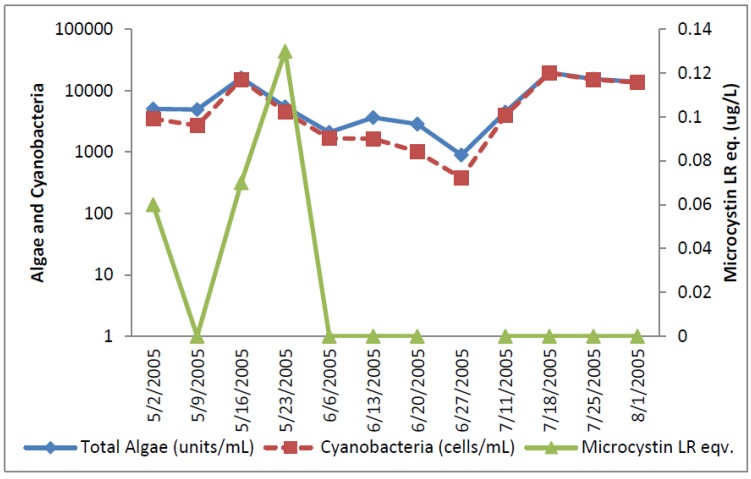
Oklahoma Total and toxic cyanobacteria and ELISA microcystin.

**Figure 10:**
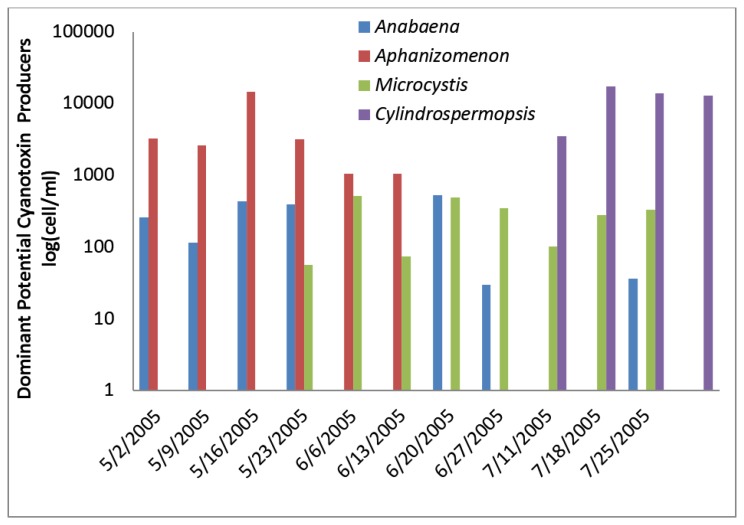
Individual toxin-producers at the Oklahoma site.

Total cyanobacteria exceeded the AL 1 10 out of 11 samples (Figure 9). *Aphanizomenon*, a potential producer of anatoxin-a and cylindrospermopsin, reached 14,600 cells/mL on 16 May 2005. *Microcystis* reached its peak of 500 cells on 20 June 2005 and accounted for 50% of the potential microcystin-producers on that day, with the remainder being *Anabaena*. *Cylindrospermopsis* reached its peak on 18 July 2005 of 17,000 cells/mL and then declined. It was the sole potential cylindrospermopsin-producer in those samples. These results shown in Figure 10 indicate that all three groups of potential toxin-producers were well represented at the Oklahoma site at some time during the study, but that cylindrospermopsin-producers reached numbers that were higher than the other types.

Microcystin as determined by ELISA was detected at very low concentrations between 0.06 and 0.13 ug/L in three samples near the start of sampling in the raw water. Microcystin-LW was detected by HPLC at a concentration of 0.9 ug/L on 13 June 2005, although it was not detected by ELISA in this sample. Cylindrospermopsin was detected by HPLC/PDA at a concentration of 0.41 ug/L on 2 May 2005. Relatively high levels of *Aphanizomenon*, 3,200 cells/mL, were found in this sample. Anatoxin-a was not detected by HPLC/PDA in any raw water sample.

The Oklahoma utility had low numbers of toxin-producing cyanobacteria in the finished water. Finished water had 46 cells/mL of *Microcystis* and 60 cells/mL of *Aphanizomenon* in the 13 June 2005 sample, 8 cells/mL of *Microcystis* in the 27 June 2005 sample, and 6 cells/mL of *Microcystis*/mL in the 1 August 2005 sample. Treatment removed between 0.2 and > 3.2 logs of toxin producing cyanobacteria for the Oklahoma distribution water (Table 1). No microcystin, cylindrospermopsin or anatoxin-a was detected in the Oklahoma distribution water by ELISA or HPLC.

#### 2.1.5. Vermont

**Figure 11:**
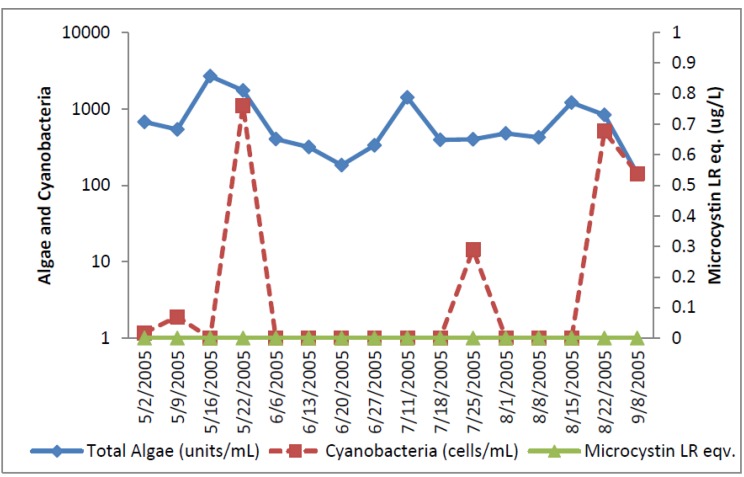
Vermont total cyanobacteria and total toxin-producers. Microcystin was not detected in any sample.

**Figure 12:**
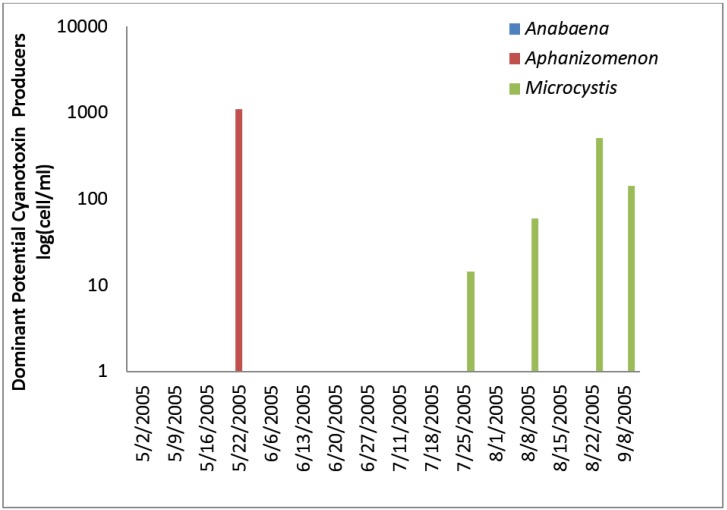
Individual toxin-producers at the Vermont site.

The total cyanobacteria never exceeded the AL 1. The total algal counts at the Vermont site reached 2600 units/mL once 16 May 2005 (Figure 11). Very low numbers of total toxin-producers were found in some samples and never approached the WHO AL 1 (Figure 12). When toxin-producers became most numerous on 22 August 2005, they only approached 500 total cyanobacteria in the sample. On this date, microcystin producers became the most numerous toxin-producer detected during sampling. No algal or cyanobacterial cells were detected in any finished water sample during sampling. Water treatment removed total cyanobacteria by as much as log 3.1 and toxin-producers by as much as log 2.2 (Table 1). Microcystin, cylindrospermopsin, and anatoxin-a were not detected in any raw water or distribution system water samples by ELISA or HPLC/PDA.

### 2.2. Fate of Cyanobacteria in Conventional DWTP

General observations for cyanobacteria included:

- The potential toxin-producing genera varied temporally and spatially between sites.
- *Microcystis* was the most geographically-distributed genera.
- High concentrations of cyanobacteria in the raw water did not lead to high concentrations of cyanotoxins in the raw or finished drinking water.
- Removal of cyanobacterial cells was very good in these five conventional DWTPS.
- It was observed that the filamentous cyanobacteria, especially *Aphanizomenon* are most likely to break through filters and be found in the finished water.

The concentration of cyanobacterial cells in raw water were not always related to concentrations of microcystins. This can be seen at the California site where the highest concentration of microcystin occurred after the highest concentration of potential microcystin-producer cells occurred. Possible factors causing this lack of relation could be that some strains of potential toxin-producing species did not have the toxin gene [29], or that microcystin genes may not be expressed at some times [30,31].

After conventional drinking water treatment, few cyanobacterial cells were found in the finished drinking water (Table 1). The log removal of total toxin producers ranged between log 1.5 and >log 5.5. When toxin-producers were found in finished drinking water, they were well below the WHO AL 1 level of concern of 2000 cells/mL in all cases.

In general these high removal efficiencies of cyanobacteria are encouraging with respect to *Microcystis* and microcystins. As long as the cells are intact and the bulk of the toxin is intracellular, conventional DWTPs should remove both cyanobacteria and cyanotoxins. Hoeger *et al*. [32] observed similar performance >1.5 log removal at a conventional DWTP in Germany. There is a further note of caution however. If a high percentage of microcystin is extracellular, there is a potential that PAC and oxidation may not provide sufficient removal of microcystins [32]. Furthermore, and somewhat in contrast to our study, Zamyadi *et al*. [33] observed *Aphanizomenon* and some *Pseudoanaebena* breaking through the filters into the finished water above the WHO AL 1 level (8800 cells/mL). Their study, which consisted of through-plant monitoring of cyanobacterial cell removal in a conventional DWTP, included intensive monitoring of the source, raw, clarifier, filter, sludge, and finished water for cyanobacterial cells. Their report and our observations of lower cell concentrations breaking through the filters, suggest that filamentous cyanobacteria, especially *Aphanizomenon* are likely to break through filters and to be found in the finished water. They observed these breakthroughs of filamentous bacteria when very high concentrations of the cyanobacterial were present in the raw water and clarifier sludge.

### 2.3. Fate of Cyanotoxins in Conventional DWTPs

Our study showed that microcystin was detected in 40 of 71 total raw water samples (56%) at less than 1 ug/L of microcystin-LR eq. Of these 40 detections, 36 were below <0.2 ug/L of microcystin-LR eq. These detections occurred in 4 of the 5 utilities sampled. Only one (1.4%) raw water sample exceeded the provisional WHO GV of 1 ug/L microcystin-LR eq. We observed no detectable microcystin in the finished water. Only one detection of cylindrospermopsin was observed in any raw water sample and none was observed in any finished water. We never observed anatoxin-a in the raw water or finished water at any DWTP in our study.

Microcystin data presented by Carmichael [22] indicated that for samples taken during bloom conditions 84% of samples contained detectable but less than 1 ug/L microcystin-LR in the raw water. Approximately 5% of the raw water samples had greater than 1 ug/L of microcystin. The remaining 11% had microcystin concentrations below detection. In contrast to our study, Carmichael observed that approximately 65% of the finished water samples contained detectable microcystin and 1% of those finished samples exceeded 1 ug/L microcystin-LR eq.

Haddix *et al*. [34] monitored 33 U.S. DWTPs collecting 206 raw water samples and 77 finished water samples. No cyanobacteria were monitored. Approximately 87% of the raw water samples had detectable MC-LR. The mean concentration was 0.307 ug/L MC-LR eq. Seven percent of the raw samples exceeded 1 ug/L MC-LR. WHO GV. Haddix *et al*. observed that 30% of their finished water samples contained detectable MC-LR. No finished water sample exceeded the WHO GV for MC-LR. The mean MC-LR eq. concentration in the finished water was 0.036 ug/L MC-LR eq.

The concentrations of microcystins in raw water reported in this work are consistent with previous studies, albeit somewhat lower given the moderately high levels of cyanobacteria present. The biggest discrepancy is in the finished water. Carmichael’s observations of increased microcystin detections in finished water as compared to our study may have been due to the higher raw water microcystin concentrations entering the plants surveyed in his study.

There are several limitations that must be considered when comparing ELISA to HPLC methods. The two methods have different detection limits with ELISA being more sensitive but less specific. The ELISA also has cross reactivities to microcystin congeners ranging from 0.35 for MC-YR to 1.0 for MC-RR relative to MC-LR. There is no cross reactivity listed for MC-LW congener and the EnviroLogix ELISA kit. This may explain the discrepancy for the Oklahoma site where MC-LW was determined by PDA to be 0.9 ug/L versus 0.13 ug/L for ELISA. In the case of the California plant where MC-LR was detected at 0.7 ug/L by PDA and 0.19 ug/L by ELISA, the anomaly is probably due to differences in the sample preparation or matrix inhibition of the ELISA. In the case of the Florida samples, where MC-LR equivalents were 1.4 ug/L and the PDA had no detection, the discrepancy probably lies in the sample preparation. At low toxin levels, discrepancies between ELISA and PDA methods can be large and care should be taken to not over interpret any single result.

### 2.4. Application of the WHO Alert Level Framework

Watzin *et al*. [35] examined the relation of the WHO Alert Level Framework to microcystin concentration for Lake Champlain in Vermont. These investigators found that an Alert Level 1 of 2000 cells/mL was conservative, and microcystin concentrations in a developing bloom did not approach 1 ug/L until the density of potential microcystin producers was greater than 4000 cells/mL. They also found that cell density was not directly correlated with microcystin concentration. They observed that of 48 samples taken that had below 4000 cells/mL of potential microcystin-producers, nine of these samples had detectable levels of microcystin. The average and median microcystin levels found in the 39 samples with detectable microcystin were 0.42 ug/L and 0.04 ug/L respectively with a maximum of 2.42 ug/L found in one sample. These results are similar to those found in our study. Watzin *et al*. [35] also found that high toxin concentrations were rare with low cell concentrations except when a bloom was breaking down. Based on the results of our study, we concur with Watzin *et al*. [35] that the WHO Alert Level 1 framework of 2000 cyanobacteria cells/mL is overly conservative. Because of the variable nature of blooms in each source water and the high cost associated with toxin sampling and analysis, it seems prudent that each DWTPs should consider developing cyanobacterial cell count action levels that trigger toxin sampling and analysis for their local conditions. Furthermore, our results suggest that the composition should be determined and different levels set for each genera. For instance the WHO AL 1 would not be appropriate for cylindrospermopsin or anatoxin-a potential producers. Our results showed that even when these genera were present at levels in excess of 2000 cells/mL anatoxin-a and was never detected and cylindrospermopsin was only detected once at a level, below most proposed GV for this toxin.

The original WHO Alert Level framework (ALF) is based on total cyanobacterial cells/mL. This management scheme provides a useful starting point but should not be arbitrarily adopted in North America. The Water Safety Plan approach should be considered as a tool to modify the WHO ALF for local conditions including Alert Levels based on cell concentrations of locally present toxin producing genera [36]. Additionally, the expected ability of particular drinking water treatment systems to remove toxins should be included in individual water safety plans. Some countries such as the Czech Republic continue to use the original WHO framework. Other countries have based their alert levels on anywhere from 5000 to 50,000 cyanobacteria cells/mL. In the case of Australia, Health Alert Levels, are based on specific species present. However, all regular interval microscopic methods have a significant disadvantage in that they cannot capture the highly dynamic changes in cyanobacterial cell concentrations. New low-cost sensors based on phycocyanin fluorescence can overcome some of the problems inherent in infrequent microscopic monitoring and can be combined with microscopy to provide a comprehensive management system that should be given consideration in the development of a site specific water safety plan [37].

## 3. Experimental Section

### 3.1. Sampling Procedures

Five drinking water utilities where chosen for this study based on a history of the occurrence of potential toxin-producing cyanobacteria and their geographic distribution across the U.S. Utilities selected were located in the states of California, Oklahoma, Vermont, Texas, and Florida. All five utilities are conventional coagulation/filtration treatment plants. The Florida utility utilized two source waters, a river and a reservoir; samples were taken from both. The general physical and chemical treatment processes used by each utility are listed in Table 2.

Each utility was sampled at two locations; the raw water (prior to any chemical addition) and the finished water (first point of distribution). Samples were collected for 12 consecutive weeks from May to August 2005 at all utilities except California, where 11 weekly samples were taken and Vermont where 16 weekly samples were taken. Samples were collected for both cyanotoxin analysis and algae or cyanobacteria identification/enumeration. Cyanotoxin samples were collected in duplicate using 1 L amber glass bottles. Approximately 100 mg/L of ascorbic acid was added to the finished water samples to inactivate any residual free chlorine. Cyanotoxin samples were refrigerated until shipped on ice via an overnight delivery service. Cyanobacterial identification/enumeration samples were collected at each sample location and placed in 125 mL amber Nalgene™ bottles containing Lugol’s reagent and mailed priority next day mail to the analysis laboratory.

**Table 2:** Utility Information.

Site Identification Number	State	Source Water	PAC	Coagulation/Flocculation	Clarification	Filtration	Disinfection
123	VT	Lake	-	x	x	Sand/Anthracite	Chlorine
485	FL	River/Reservoir	x	x	x	x	Chloramines
619	OK	Reservoir	x	x	x	Sand/Anthracite	Chlorine
762	CA	Reservoir	-	x	x	Sand/Anthracite	Ozone/Chloramines
929	TX	Reservoir	x	x	x	Sand/Anthracite	Chloramines

### 3.2. Sample Preparation and ELISA Analysis

An unfiltered 1 mL aliquot from both the raw and finished sample from each location was sonicated at 60 watts for 5 min. The Envirologix Quantiplate™ ELISA (enzyme-linked immunosorbant assay) Kit for total microcystins was employed as a screen for microcystin. The ELISA kit’s high sensitivity option found in the manufacturer’s directions, was used to quantify microcystin concentrations from 0.05 to 0.83 ug/L. Samples were analyzed in duplicate. Samples below 0.05 ug/L were reported as <0.05 ug/L and samples above 0.8 ug/L were repeated at 1:10, 1:100, and 1:1000 dilutions. The quality control program for the ELISA analysis consisted of a laboratory sample duplicate, laboratory fortified sample matrix and continuing calibration verification standard analyzed every seven samples. Analyses were acceptable if the quality control samples were within 15 percent of the expected value. If any value fell outside of the acceptance criteria the batch was reanalyzed.

### 3.3. Sample Preparation & HLPC-PDA Analysis

One liter of the cyanotoxin sample was filtered through a Whatman^®^ Glass microfiber filter. The filters and 250 mL of the filtrate were frozen at −80 °C and archived until analysis. The archived glass-fiber filter was homogenized in 4 mL of 50% methanol. The homogenized sample was sonicated in an ultrasonic bath at 60 watts for 25 min and centrifuged for 5 min at 11,000 rpm. The supernatant was collected and the pellet was extracted by two sequential 2 mL 85% methanol extractions. Additional sonication and centrifugation was performed between extractions. All supernatants were pooled together and brought to dryness under nitrogen. Samples were reconstituted to a final volume 1 mL in 20% methanol. The filtrate was lyophilized then extracted by two sequential 2 mL 85% methanol extractions. All supernatants were pooled together and brought to dryness under nitrogen. Samples were reconstituted to a final volume 0.25 mL in 20% methanol.

Samples were analyzed for cylindrospermopsin, anatoxin-a, and microcystin-RR, LR, LA and LF using a High Performance Liquid Chromatograph with a Photodiode Array (HPLC/PDA) [38]. A standard for microcystin-YR, identified as a high priority congener by EPA, was not available and it was therefore not analyzed. Samples below the detection limit of 0.25 ug/L were reported as <0.25 ug/L and samples above the 2 ug/L were repeated at 1:10, 1:100, and 1:1000 dilutions.

**Figure 13:**
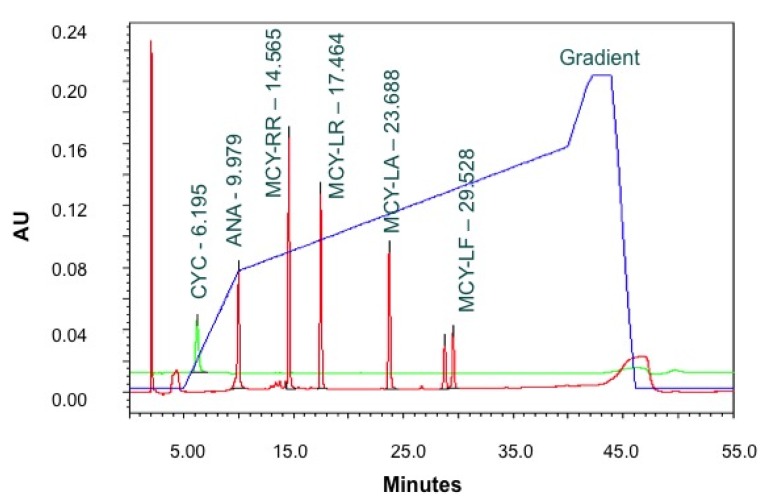
Chromatograph of HPLC/PDA Run.

The HPLC/PDA method used a 0.02% trifluoroacetic acid (TFA) acetonitrile and 0.02% TFA water gradient. The 55-min chromatographic run utilized a 5-min 2% acetonitrile isocratic period followed by a 35%, 70%, and 90% acetonitrile gradient (Figure 13). The toxins were separated on a C18 column (Atlantis^®^, Waters, Milford, MA, USA) in the following order; cylindrospermopsin, anatoxin-a, microcystin RR, LR, LA, and LF. Anatoxin-a was monitored at a wavelength of 227 nm and the microcystins were monitored at a wavelength of 238 nm, cylindrospermopsin was monitored at 261 nm. (Table 3) The results of the filter extract (intracellular cyanotoxins) and lyophilized filtrate (extracellular cyanotoxins) were combined to provide a total cyanotoxin concentration. The quality assurance and quality control consisted of running a sample blank, a duplicate, a fortified duplicate, and a positive control every 10 samples. The acceptance criteria for these analyses were relative errors less than 15% for duplicate and positive controls and relative recovery within 20% for the fortified duplicate.

**Table 3:** HPLC/PDA Analyte Parameters.

Cyanotoxin	Retention Time (min)	Wavelength (nm)	Method Detection Limit (ug/L)
Cyclindrospermopsin	6.195	261	0.25
Anatoxin-a	9.979	227	0.25
Microcystin-RR	14.565	238	0.25
Microcystin-LR	17.464	238	0.25
Microcystin-LW	29.528	238	0.25

### 3.4. Cell Counts by Microscopy

Twenty-five milliliter water samples were settled in Utermohl plankton sedimentation chambers for at least 24 h. A qualitative and quantitative count was performed at 200X using an inverted phase-contrast microscope. Cyanobacteria and algal identification were made using standard taxonomic references, such as Prescott [39]. A minimum of 300 cyanobacterial units or 100 microscope fields were counted per sample. This approach will yield an estimate of 10–20 percent error for the dominant genera, and 20–60 percent for the subdominant genera [5]. The cyanobacteria observed in this study grew as filaments or colonies consisting of a large numbers of cells and counted as cell-aggregations (units), rather than as individual cells. The number of cells per unit varied substantially by genera, site and sampling date. For each sample the average number of cells / unit was determined by counting individual cells/units in ten fields. Total cyanobacteria and potential toxin-producing cyanobacteria counts are reported. Because of the variable number of cells per unit for different genera and our ability to quantify 1–2 units/mL, low cell concentrations are highly variable and may not be statistically significant. The plots of specific potential toxin producers at each site were based on the following genera; microcystin- *Microcystis*, *Oscillatoria*, *Nostoc*, *Hapalosiphon*, *Anabaenopsis*, and *Anabaena*; cylindrospermopsin- *Aphanizomenon* and *Cylindrospermopsis*; anatoxin-a- *Aphanizomenon*, *Anabaena*, and *Oscillatoria*.

## 4. Conclusions

The presence of toxic cyanobacteria and microcystin in drinking water source waters is a widespread phenomenon across the U.S. Even though the concentrations of cyanobacterial cells were elevated and in many cases exceeded the WHO AL 1 cell limit of 2000 cells/mL, microcystin concentrations were low and only exceeded the WHO provisional guidance value, 1 ug/L, once. Furthermore no anatoxin-a was measured above the detection limit at any site. Cylindrospermopsin was detected once. More important, conventional treatment effectively removed most toxin producing cyanobacterial cells and toxins at levels observed in this study. When our results are combined with previous studies it emphasizes the highly variable nature of the cyanobacteria problem. The WHO AL framework is conservative with respect to the levels of cyanobacterial cells that trigger increased monitoring. It should be considered a starting point and the cyanobacterial cell levels adjusted upwards as necessary to reflect local conditions that will balance the available resources for monitoring and consumer safety.
